# Multidisciplinary Approach to Melanoma: A Case Report on Below-Knee Amputation and Lymph Node Dissection

**DOI:** 10.7759/cureus.65793

**Published:** 2024-07-30

**Authors:** Rishika Bhatnagar, KM Hiwale

**Affiliations:** 1 Pathology, Jawaharlal Nehru Medical College, Datta Meghe Institute of Higher Education and Research, Wardha, IND

**Keywords:** dermatofibroma, hypertension, diabetes mellitus, melanoma, inguinal lymph node dissection, below-knee amputation

## Abstract

A 65-year-old male presented with progressive swelling and difficulty in walking due to a right foot sprain. Initial treatments were conducted in Chandrapur, followed by referral to Acharya Vinoba Bhave Rural Hospital for further evaluation and management. The patient, a known case of diabetes mellitus and hypertension, reported an insidious onset of right foot swelling over two months. A physical examination revealed stable vital signs; no significant abnormalities were observed during the systemic examination. Laboratory investigations indicated mild anemia and slightly elevated liver enzymes. Imaging studies, including MRI and CT scan, identified an ill-defined lesion on the medial aspect of the right foot, consistent with dermatofibroma. The patient underwent a below-knee amputation with inguinal lymph node dissection on 31st May 2024. The procedure, performed under spinal and epidural anesthesia, involved meticulous dissection and ligation, with the posterior flap sutured using Ethilon 2-0 (Ethicon, Cincinnati, OH, USA). Post-operative management included IV antibiotics and supportive care. The patient’s postoperative course was uneventful, with a healthy suture line and stable vitals upon discharge. Histopathological evaluation of the resected specimen confirmed melanoma, with immunohistochemistry revealing HMB-45 and S-100 negativity. The patient was discharged with advice on local hygiene, physiotherapy, dietary recommendations, and a follow-up schedule. This case highlights the importance of comprehensive multidisciplinary management in treating malignancies complicated by chronic conditions. Early diagnosis, appropriate surgical intervention, and diligent post-operative care are crucial for favorable outcomes in complex oncological cases.

## Introduction

Melanoma, a malignant tumor of melanocytes, is one of the most aggressive forms of skin cancer. It often presents as a new or changing lesion on the skin and has the potential for early metastasis, making timely diagnosis and management crucial for patient outcomes [1]. Although melanoma accounts for a small percentage of skin cancer cases, it is responsible for the majority of skin cancer-related deaths worldwide [2]. The incidence of melanoma has been increasing globally, with substantial variations in survival rates depending on early detection and the implementation of effective treatment strategies [3].

Dermatofibroma, also known as benign fibrous histiocytoma, is a common benign skin tumor. Although typically harmless, dermatofibromas can occasionally be misdiagnosed as malignant due to their clinical and histological similarities with more aggressive tumors [4]. Accurate diagnosis is essential to distinguish between benign and malignant lesions and to guide appropriate treatment.

This case report details a 65-year-old male who presented with progressive swelling and difficulty in walking due to a right foot sprain. The initial diagnosis of dermatofibroma was complicated by the discovery of melanoma, necessitating a below-knee amputation with inguinal lymph node dissection. This case underscores the importance of comprehensive evaluation and multidisciplinary management in complex cases involving malignancies and chronic conditions.

## Case presentation

A 65-year-old male presented to tertiary health care in central India with a primary complaint of swelling in his right foot, which had persisted for two months. The patient initially noticed the swelling following a sprain in his right foot due to trauma. The swelling was progressive and insidious in onset, accompanied by difficulty walking. He sought primary treatment in Chandrapur before being referred to tertiary health care in central India for further management. The patient's medical history included known cases of diabetes mellitus and hypertension, for which he was on regular medication. Additionally, he had undergone a hemorrhoidectomy in 2017. On physical examination, his vital signs were stable: pulse 88/min, respiratory rate 18/min, blood pressure 110/70 mmHg, and he was afebrile. A general examination revealed no significant abnormalities. Systemic examination showed bilateral air entry in the respiratory system, normal heart sounds, and no neurological deficits. The abdominal examination was normal, and the musculoskeletal examination showed no gross abnormalities. Patient laboratory values are described in Table 1.

**Table 1:** Patient’s vitals and laboratory results RBC: red blood cell, INR: international normalised ratio

Parameter	Value	Reference Range
Pulse	88/min	60-100/min
Respiratory Rate	18/min	12-20/min
Blood Pressure	110/70 mmHg	90-120/60-80 mmHg
Temperature	Afebrile	36.5-37.5°C
Hemoglobin	5.6 g/dL	13.8-17.2 g/dL
RBC Count	3.02 x 10^6/µL	4.7-6.1 x 10^6/µL (M)
INR	1.42	0.8-1.1
Random Blood Glucose	116 mg/dL	70-140 mg/dL

**Figure 1:**
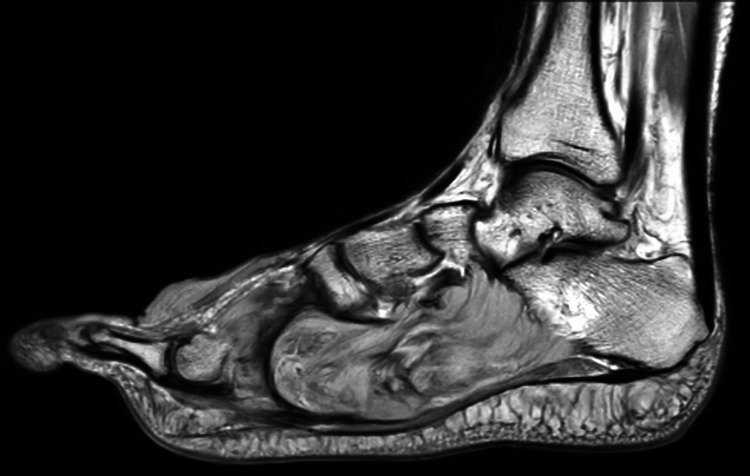
Magnetic resonance imaging (MRI) revealed an ill-defined lesion along the medial aspect of the right foot extending to the plantar region

Imaging studies, including an MRI, revealed an ill-defined lesion along the medial aspect of the right foot extending to the plantar region, consistent with a previously diagnosed dermatofibroma (Figure 1).

**Figure 2:**
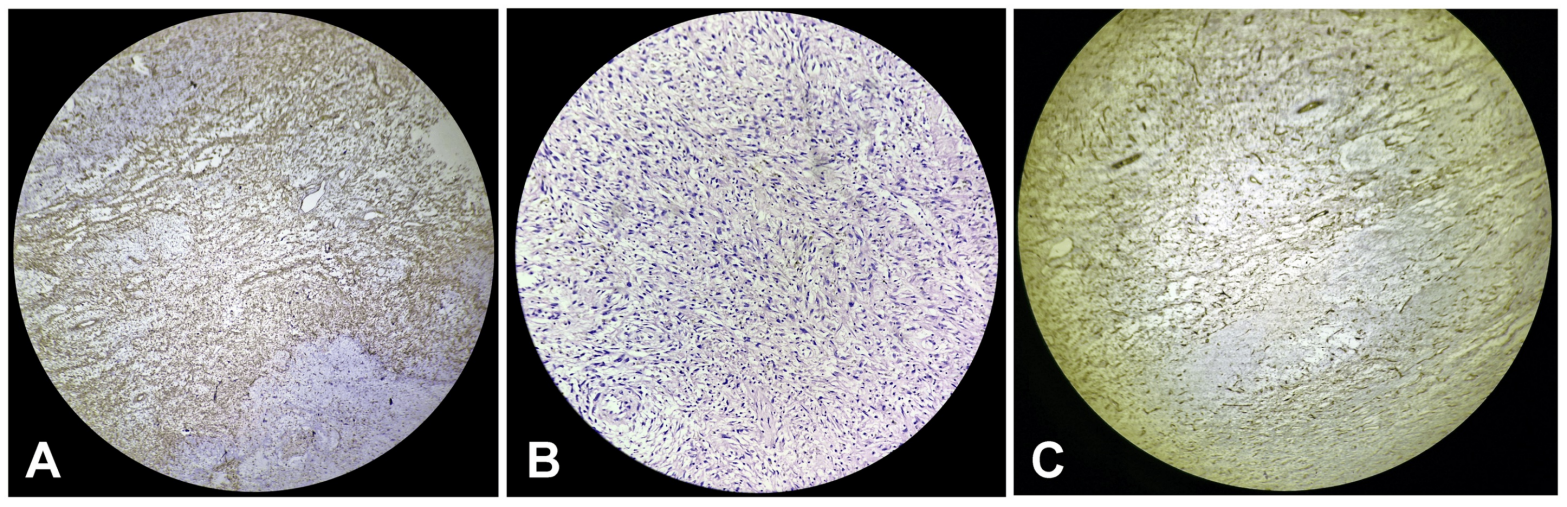
A) H & E section shows abundant eosinophilic cytoplasm, nuclei show pleomorphism and are ovoid to elongated in shape arranged in a storiform pattern, B) Immunohistochemistry section is positive for vimentin, C) Immunohistochemistry section is negative for CD34

The patient underwent a below-knee amputation of the right lower limb with inguinal lymph node dissection. The surgery was performed under spinal and epidural anesthesia. The procedure involved marking an anterior skin incision 10 cm below the tibial tuberosity, followed by a meticulous dissection layer by layer, ligating the anterior tibial pedicle, and achieving hemostasis. The bones were cut using a jiggly saw, and the inguinal lymph nodes were dissected. Post-operatively, a Romovac drain was placed, and the posterior flap, comprising the posterior fascia and gastrocnemius, was sutured using Ethilon 2-0 (Ethicon, Cincinnati, OH, USA). The patient's postoperative course was uneventful. The patient received intravenous antibiotics consisting of ceftriaxone at a dose of 2 grams every 12 hours and metronidazole at 500 milligrams every eight hours. For pain management, morphine was administered at a dose of 2 milligrams intravenously every four hours as needed, and ketorolac was given at 30 milligrams intravenously every six hours as required for pain relief. The drain was removed on the fourth post-operative day following a decrease in output. His suture line remained healthy without any signs of infection or wound gape. He was discharged with stable vitals and was advised to maintain local hygiene, perform regular physiotherapy exercises, and follow a high-protein diet. The patient was instructed to abstain from alcohol and smoking and to follow up in the surgery outpatient department after seven days with his histopathology reports. Histopathological evaluation of the resected specimen confirmed the diagnosis of melanoma, with immunohistochemistry showing HMB-45 and S-100 negativity (cytoplasmic positivity only). Given the comprehensive care and monitoring, the patient's condition was stable at discharge, highlighting the importance of multidisciplinary management in complex malignancies and chronic conditions (Figure 2).

## Discussion

This case highlights the complexities involved in managing a patient with a history of diabetes mellitus and hypertension who presented with a malignant melanoma of the foot, ultimately necessitating a below-knee amputation and inguinal lymph node dissection. The patient’s history of progressive swelling following trauma underscores the importance of thorough evaluation and timely referral in cases where initial conservative management fails to resolve the symptoms. Melanoma of the foot is a rare and challenging condition due to its atypical presentation and frequent delay in diagnosis [5]. Foot melanoma accounts for approximately 3-15% of all cutaneous melanomas and often presents at a more advanced stage compared to melanomas at other sites due to its location and the potential for misdiagnosis as benign conditions such as dermatofibroma or plantar warts [6,7]. In this case, the patient initially received a dermatofibroma diagnosis, highlighting the diagnostic challenge and the need for vigilance and biopsy in persistent or atypical lesions.

The decision to perform a below-knee amputation in this patient was based on the extent of the lesion and the involvement of underlying structures, as confirmed by MRI and CT imaging [8]. Surgical management of foot melanoma often necessitates wide local excision or amputation to achieve clear margins, especially in cases with deep tissue involvement [9]. The inguinal lymph node dissection was also a critical component of the surgical plan, given the propensity for melanoma to metastasize to regional lymph nodes. The histopathological evaluation confirmed the diagnosis of melanoma, with immunohistochemistry showing negativity for HMB-45 and S-100, which are commonly used markers in melanoma diagnosis. The negative staining for these markers, in this case, may suggest a unique or variant presentation of melanoma, as typical melanomas usually exhibit positivity for these markers [10]. This highlights the importance of a comprehensive histopathological and immunohistochemical evaluation in diagnosing melanoma.

Post-operative care is crucial in managing patients who undergo major amputations. In this case, the patient received intravenous antibiotics, analgesics, and supportive treatment, contributing to an uneventful recovery. Regular monitoring and timely drain removal helped prevent post-operative complications such as infection and seroma formation. The patient’s stable condition at discharge and the healthy suture line indicate the effectiveness of the post-operative care protocol followed. The multidisciplinary approach involving surgical oncology, pathology, radiology, and supportive care teams was instrumental in successfully managing this complex case. The patient’s known comorbidities of diabetes and hypertension added another layer of complexity, necessitating careful management to prevent complications related to these conditions.

## Conclusions

This case highlights the complexities of diagnosing and managing this rare condition, especially in the presence of comorbidities like diabetes and hypertension. The initial misdiagnosis of dermatofibroma underscores the need for vigilance and biopsy in persistent lesions. Comprehensive surgical intervention, including below-knee amputation and inguinal lymph node dissection, along with meticulous post-operative care, led to a successful recovery. This case emphasizes the importance of early and accurate diagnosis, appropriate surgical management, and coordinated multidisciplinary care in achieving favorable outcomes for patients with complex oncological conditions.
